# Rhein laden pH-responsive polymeric nanoparticles for treatment of osteoarthritis

**DOI:** 10.1186/s13568-020-01095-3

**Published:** 2020-08-31

**Authors:** Bo Hu, Feng Gao, Chunbao Li, Boqing Zhang, Mingyang An, Ming Lu, Yufeng Liu, Yujie Liu

**Affiliations:** 1 Department of Orthopedic, Beijing Chaoyang Integrative Medicine Emergency Medical Center, Beijing, 100022 China; 2 Medical School of Chinese People’s Liberation Army, Beijing, 100853 China; 3 National Institute of Sports Medicine, Beijing, 100061 China

**Keywords:** PLGA, Nano-carriers, pH-sensitive, Anti-inflammatory, Drug delivery

## Abstract

Osteoarthritis (OA) is a condition associated with severe inflammation, cartilage destruction and degeneration of joints. Rhein (Rh) is an effective anti-inflammatory drug with proven efficacy in in-vitro and in-vivo models. pH sensitive Rh and NH_4_HCO_3_ laden poly (lactic-co-glycolic acid (PLGA) nanoparticles (NPs) (Rh-PLGA-NPs@NH_4_) are developed for an effective treatment of OA. The Rh-PLGA-NPs@NH_4_ are prepared along with Rh-PLGA-NPs as a control by double emulsion method. Rh-PLGA-NPs@NH_4_ was characterized for their size, shape, morphology and encapsulation efficiency (EE). The effect of pH on release of Rh from Rh-PLGA-NPs@NH_4_ was studied at different pH. Further, the cytotoxicity effect of Rh-PLGA-NPs@NH_4_ on THP-1 cells were evaluated. Anti-inflammatory efficacy was evaluated on LPS stimulated THP-1 cells and the release of pro-inflammatory cytokines was evaluated and compared with control. The size of Rh-PLGA-NPs@NH_4_ and Rh-PLGA-NPs was found to be 190.7 ± 1.2 nm and 134.6 ± 2.4 nm respectively with poly dispersity (PDI) 0.14 and 0.15. The zeta potential of Rh-PLGA-NPs@NH_4_ was found to be -22 ± 1.12 mV. Rh-PLGA-NPs@NH_4_ were uniform, smooth and spherical shape as confirmed using electron microscopy analysis. Rh-PLGA-NPs@NH_4_ release the Rh more effectively in the low pH of synovial fluid environment (SFE). Rh-PLGA-NPs@NH_4_ also significantly affect inflammatory cytokines TNF-α and IL-1β and reduced their release in LPS stimulated THP-1 cells. Reactive oxygen species (ROS), a mediator responsible for the cartilage collapse was also found to be reduced. Results proposes that Rh-PLGA-NPs could provide therapeutic solution to those patients who suffer from chronic joint ailments by reducing the progression of OA.

## Introduction

One of the most prevalent disorders associated to joints is osteoarthritis (OA), which mainly affects the hand, hip and knee joints. (Zerrillo et al. 2019). Mostly in osteoarthritis osteophyte formation, sub-chondral bone sclerosis, degeneration of articular cartilage, synovial inflammation occurs (Buckland-Wright 2004; Castaneda et al. 2012). However, it is assumed that tumor necrosis factor (TNF-α) and pro-inflammatory cytokine factor (IL-1β) takes main role in the development of OA (Ashford et al. 2014; Wojdasiewicz et al. 2014; Roy et al. 2015).

The transformation in OA therapy, which includes cartilaginous matrix precursors, cytokine modulators etc., leads to the advancement in metabolic activity of cartilage. The derivatives of anthraquinone such as diacerein have acquired attention of scientist and researchers for many years due to its reticence effect on pro-inflammatory cytokine interleukin 1β action (Qvist et al. 2008). Diacerein has been approved for clinical trials, but—due to low water solubility, low bioavailability and adverse reactions including liver toxicity and diarrhea, it is not in practice (Louthrenoo et al. 2007; Fidelix et al. 2014). Rh is the most popular diacerein metabolite, which inhibit the production of ROS and significantly reduce the IL-1β and nitric oxide (NO) production and protects the cartilage degeneration (Legendre et al. 2007; Wojdasiewicz et al. 2014). To deliver and target the therapeutic agents, nanoparticles (NP) with various advantages are widely used. NPs are composed of polymers or lipids that are biocompatible and biodegradable, with great potential for targeted therapies (Kiran et al. 2020). A FDA approved polymer in drug delivery is Polylactic-co-glycolic acid (PLGA) and it is known for its degeneration property in water (Shive et al. 1997; Ulery et al. 2011; Dwivedi et al. 2019). Both hydrophilic and hydrophobic compounds could be loaded in the polymeric NPs (Ulery et al. 2011).

In the present study, we aim to analyze the pH influence on NPs for the treatment of OA. In addition, NH_4_HCO_3_ was selected and co-loaded in NPs along with Rh, in order to exploit the difference in the pH of synovial fluid (Farr et al. 1985). Interestingly, the porous nature of PLGA-NP allows small molecules such as H_2_O and H_3_O^+^ inside the NPs, enhancing the release of Rh. Hydrogen ions (H_3_O^+^) present at high concentrations and at low pH, react with NH_4_HCO_3_ in PLGA-NP to induce pH neutralization and result in the production of NH_4_^+^, CO_2_ and H_2_O (Zerrillo et al. 2019). This conversion caused damage to the PLGA-NPs structure with sudden release of cargo. Recently, Li et al. (2016) follow the same protocol and successfully generate a pH-responsive PLGA-NP and applied for intracellular infectious diseases treatment and viral infections.

## Materials and methods

Poly (lactic-co-glycolic acid) (PLGA, 50:50), ammonium bicarbonate (NH_4_HCO_3_) and dichloromethane (DCM) were obtained from Sigma-Aldrich (USA). DMEM and FBS were obtained from Sigma-Aldrich (USA). The chemicals obtained and reagents were of analytical grade and materials procured were utilized without further purification. The triple distilled water obtained from Millipore Milli-Q (Bedford, MA, USA) is used wherever required.

### Rational design of NPs

**Fig. 1:**
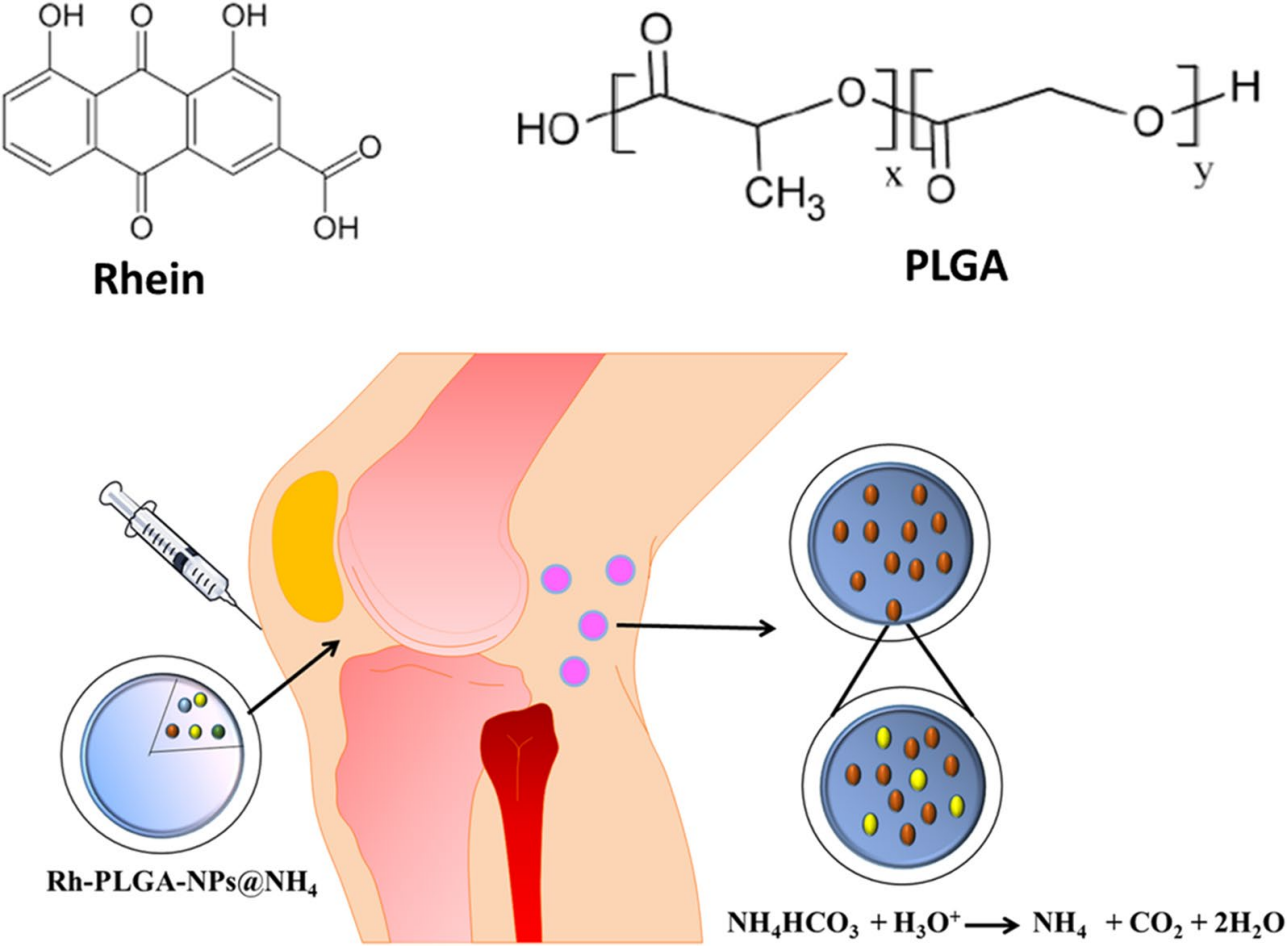
Schematic illustration of pH-responsive Rh-PLGA-NPs@NH_4_ used for the treatment of OA

A detailed design of pH-responsive Rh-PLGA-NPs@NH_4_ is elaborated in Fig. 1. The nanoparticles (NPs) were fabricated by using emulsion-solvent evaporation protocol and were encapsulated with Rh, NH_4_HCO_3_. The main aim of the addition of NH_4_HCO_3_ was to obtain a pH-responsive burst release of Rh from Rh-PLGA-NPs@NH4. The H_3_O^+^ molecules in SF with low pH, infiltrate the porous shell of Rh-PLGA-NPs and interact with encapsulated NH_4_HCO_3_ in the particles. As a result NH^4+^ ion is generated along with H_2_O and CO_2_, rupturing the shell of NPs and causing the burst release of entrapped Rh.

### Preparation of PLGA NPs loaded with Rh (Rh-PLGA-NPs)

The solvent emulsion–evaporation procedure was applied for the synthesis of PLGA NPs loaded with Rh (Rh-PLGA-NPs) (Zerrillo et al. 2019). Firstly the 2.5 mL of methanol solution having 10 mg of Rh was prepared, which was further added to the solution of PLGA (200 mg) in DCM. The organic solution was emulsified by 20 mL of acidified 0.5% w/v PVA aqueous solution. This pre-emulsion was carefully homogenized with a homogenizer system to obtain micro emulsion for 40 s at 9000 rpm. By applying gentle agitation, the organic phase was evaporated and pure NPs suspension was thoroughly rinsed with MilliQ water. Rh-PLGA-NPs were obtained from the suspension by centrifugation at 14,000 rpm and 4 °C for approximately 30 min following the lyophilisation. The particles were lyophilized using a LGJ-10 freeze dry system (Beijing, China). Blank NPs (PLGA-NPs) were also prepared by avoiding the addition of Rh in the above described method.

### Rh-PLGA-NPs synthesis with ammonium bicarbonate

To study the environment pH response of Rh-PLGA-NPs, pH-responsive NPs containing NH_4_HCO_3_ (Rh-PLGA-NPs@NH_4_) were also prepared following the double emulsion process (Castaneda et al. 2012). The aqueous phase with 5 mg of NH_4_HCO_3_ was added drop wise in an organic solvent containing PLGA. The resulting w/o emulsion was homogenized for 5 min and was then added drop wise to the 0.5% of PVA solution with stirring. The resulting w/o/w emulsion was further homogenized at 25,000 rpm. The obtained NPs were magnetically stirred for 12 min at moderate speed for complete evaporation of organic phase. The NPs were collected by ultracentrifugation at the speed of 12,000 rpm for 40 min at 4 °C. The resulting NPs were rinsed with Milli Q water in order to get rid of excess of PVA. The finally obtained NPs were again suspended in Milli Q water and lyophilized. Blank NPs (PLGA-NPs@NH_4_) were also prepared by avoiding the addition of Rh in the above described method.

### Characterization of Rh-PLGA-NPs and Rh-PLGA-NPs@NH4

The prepared particles including Rh-PLGA-NPs and Rh-PLGA-NPs@NH_4_ and Blank-NPs were characterized for their size, size distribution, polydispersion index and zeta potential. The analysis was conducted using DLS measurement at 25 °C with Zetasizer, Nano ZS series (Malvern Instruments Corp, UK). All the experimental studies were carried out in triplicate.

Further, the morphology of the Rh-PLGA-NPs including shape and size was detected by SEM (JSM-6700F). The samples were coated with gold and were analyzed at 20 kV accelerating voltage. The TEM was used for morphological analysis of Rh-PLGA-NPs using TEM (JEM 2100 LaB6, JEOL, and Japan). The diluted NPs were used and were positioned on a carbon-coated copper grid (200-mesh) and stained with phosphotungstic acid (2% PTA) solution, and desiccated at room temperature.

### Estimation of percent entrapment efficiency (%EE)

The amount of Rh encapsulated in PLGA-NPs, 5 mg of Rh-PLGA-NPs/ Rh-PLGA-NPs@NH_4_ were dissolved in methanol. Subsequently, the solution was centrifuged and the supernatants were collected and estimated using High Pressure Liquid Chromatography (HPLC). Reverse phase HPLC method was used to calculate the amount of Rh. The sample (20 µL) was injected in the HPLC having RP C-18 column and was analyzed at 25 ^o^C. The mobile phase was 2% acetic acid solution and methanol used in a ratio of 20:80 with 1 mL/ min flow rate. Rh was analyzed at a wavelength of 257 nm (Gómez-Gaete et al. 2017). The percentage drug loading (DL) and % EE of the NPs was obtained by following Eqs. 1 and 2:

1 $$\%EE=\left[\frac{Wt-Wf}{Wt}\right]\times 100$$

where, Wt = weight of total drug and W_f_ = weight of un-entrapped drug

2 $$\% DL=\left[\frac{W1}{W2}\right]\times 100$$

where, DL = Drug loading, W_1_ = amount of Rh in NPs and W_2_ = amount of NPs.

### In vitro release study of Rh-PLGA-NPs and Rh-PLGA-NPs@NH_4_.

The in vitro release analysis of Rh from free Rh, Rh-PLGA-NPs and Rh-PLGA-NPs@NH_4_ were carried out in phosphate saline buffer (PBS, pH 7.4, 5.0) and simulating mixture of the synovial fluid environment (SFE) in OA by dialysis bag diffusion method. Briefly, Rh-PLGA-NPs and Rh-PLGA-NPs@NH_4_ having 5 mg equivalent Rh were dispersed in different dialysis bag having 2 mL of dissolution medium. Further, the dialysis bag were immersed in the 500 mL of dissolution medium maintained at temperature 37 ± 0.5 °C. 1 mL sample was withdrawn at a predetermined time intervals from each dissolution mediums and was replenished to sustain the condition of sink. The released Rh concentration was analyzed by using HPLC. All the studies were performed three times and mean value was calculated (Dwivedi et al. 2014).

### Cell cultures

Human chronic myeloid leukemia cell line like THP-1 was obtained from China Center for Type Culture Collection (CCTCC) and were cultured at 37 °C, 5% CO_2_ in RPMI 1640 with 10% FBS (100 U/mL) and 1% penicillin/streptomycin mix (100 mg/mL) respectively. The unattached cells were removed, and adhered cells were rinsed with RPMI 1640 medium. The cells were counted using haemocytometer and were plated in a 96 well plate according to the need of experiment.

### Cell viability assay

THP-1 cells (1 × 10^4^ cells/ well) plated in a 96 well plate, were treated with PLGA-NPs, PLGA-NPs@NH_4_, Rh-PLGA-NPs and Rh-PLGA-NPs@NH_4_ at different concentrations. Following treatment, the cells were incubated for 24 h 37 °C in a CO_2_ furnished incubator. Afterwards, MTT (12 mM) was added to each treated well and again the cells were incubated for 3 h. The cell culture medium was removed subsequently, and 100 µL SDS-HCl mixtures were added in each well to solubilize the purple formazan precipitate for 10 min in dark. (Dwivedi et al. 2014). The absorbance was measured at 570 nm on a Bio-Rad 450 micro-plate reader. The viability of cells was calculated as:

3 $$\% CV=\left[\frac{A1}{A2}\right]\times 100$$

where, CV = Cell viability, A1 = absorbance of sample and A2 = absorbance of control.

### Rh-PLGA-NPs@NH_4_ uptake studies using flow cytometer analysis and confocal microscope.

The THP-1 cells were cultured as stated above for cell uptake studies. For uptake studies, Rh-PLGA-NPs@NH_4_ particles and other particles were encapsulated with Nile red for fluorescent tagging. Further, for flow cytometer analysis, the cells 5 × 10^5^ cells/well were seeded in a 6 well plate. Following 24 h, the developed particles were incubated with the cells for 4 h and free particles were removed from the wells by rinsing them with PBS. Subsequently, the cells were trypsinized and collected in FACS buffer for further analysis using flow cytometer analysis (Flow cytometer BD LSR-II).

The confocal uptake studies were carried out in a glass bottom culture dish in which we seeded 1 × 10^4^ cells. After 24 h, the developed particles were incubated with the cells for 4 h followed by removal of the free particles from the cell culture media by washing them with PBS. The cells were further treated with formalin for 20 min following treating them with 0.2% PBS-Triton for 20 min and finally the blocking them with 5% PBS-BSA for 30 min. The cell’s nucleus were stained with 100 ng/ mL DAPI. Subsequently, confocal microscope was used to acquire the images.

### Measurement of reactive oxygen species (ROS) generation assay

The ROS was estimated by fluorescent dye 2’7’-Dichlorofluorescin diacetate (DCFDA). The cells seeded and incubated in a 6-well plate at a density of 1 × 10^6^ cells/ well for 24 h. Afterwards, the cell were treated with control and Rh-PLGA-NPs@NH_4_. Following incubation, the cells were rinsed with PBS and collected using trypsin. The cells were re-dispersed in PBS containing 10 µM of DCFDA in dark. After 30 min the cells were directly analyzed with flow cytometer analysis (FACS, Caliber, BD Biosciences, USA). Data were evaluated at a 488 nm laser was used for excitation. Hydrogen peroxide treated cells used as (+ ve) positive control.

**Anti-inflammatory cytokine assay interleukin- 1β (IL-1β) and tumor necrosis factor alpha (TNF-α)** .

Next, we evaluated the effect of Rh-PLGA-NPs and Rh-PLGA-NPs@NH_4_ on IL-1β and TNF-α cytokines after they were incubated with LPS stimulated THP-1 cells. The cells were incubated at various equivalent concentration ranges of Rh (0–20 µM). Following 24 h of treatment, the supernatant were collected and treated with ELISA kits (Sino Biological Inc Beijing, China) to calculate cytokine concentrations for IL-1 and TNF-α using micro-plate reader and the optical density of every individual wells was calculated at 450 nm.

### Statistical analysis

The statistical analysis was achieved by 2-way analysis of variance (ANOVA) test with the help of Prism 6 software (Graph Pad, USA).

## Results

### Rh-PLGA-NPs@NH4 synthesis and characterization

**Fig. 2:**
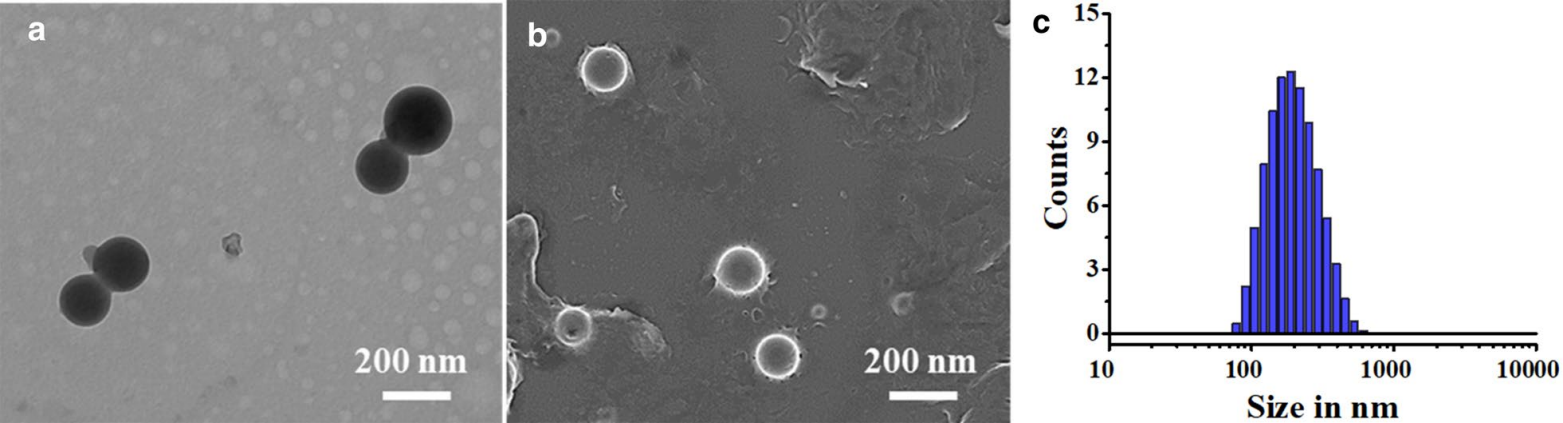
**a** TEM analysis of Rh-PLGA-NPs@NH_4_, **b** SEM analysis of Rh-PLGA-NPs@NH_4_ **c** Size distribution graph of Rh-PLGA-NPs@NH_4_

The pH-responsive, Rh and NH_4_HCO_3_ loaded PLGA-NPs (Rh-PLGA-NPs@NH_4_) were prepared exploiting the double emulsion method along with only Rh loaded Rh-PLGA-NPs. The method allows to entrap Rh into the outer shell of the NPs. The results of mean particle size of Rh-PLGA-NPs@NH_4_, Rh-PLGA-NPs along with their zeta potential and %EE have been summarized in Table 1. The Rh-PLGA-NPs@NH_4_ had a particle size and polydispersity index (PDI) more than Rh-PLGA-NPs. The size of Rh-PLGA-NPs@NH_4_ and Rh-PLGA-NPs was found to be 190.7 ± 1.2 nm and 134.6 ± 2.4 nm respectively with poly dispersity (PDI) 0.14 and 0.15. The zeta potential of Rh-PLGA-NPs@NH_4_ and Rh-PLGA-NPs were negative and was found to be − 22 ± 1.12 mV and − 16 ± 1.13 mV respectively. % EE of Rh-PLGA-NPs@NH_4_ and Rh-PLGA-NPs was found to be 46.7 ± 0.10 and 64.4 ± 2.11 respectively representing a slightly lower % EE for Rh-PLGA-NPs@NH_4_ which probably due to the presence of NH_4_HCO_3_. The size range of all developed NPs were optimum which could be actively taken into cells through endocytosis methodology (Shang et al. 2014). The appropriate negative zeta potential of Rh-PLGA-NPs@NH_4_ and Rh-PLGA-NPs indicates medium to high NPs stability (Ayala et al. 2013; Bhattacharjee 2016). The Rh-PLGA-NPs@NH_4_ morphology confirmed by SEM and TEM studies revealed uniformly distributed smooth surface spherical particles. Figure 2a, b represents the TEM and SEM images of Rh-PLGA-NPs@NH_4_ respectively whereas Fig. 2c represents the size distribution graph of Rh-PLGA-NPs@NH_4_. In summary, the above results conclude that the NPs produced are safe and appropriate for in vitro experiments.

**Table 1:** Particle size, poly dispersity index, zeta potential and % EE of different Lip

Formulation	Size (nm)	Poly dispersity index (PDI)	Zeta-potential (mV)	% Entrapment Efficiency (± SD)
PLGA-NPs	116.8 ± 1.5	0.12 ± 0.04	− 6.8 ± 1.27	–
Rh-PLGA-NPs	134.6 ± 2.4	0.15 ± 0.013	− 16 ± 1.13	64.4 ± 2.11
Rh-PLGA-NPs@NH_4_	190.7 ± 1.2	0.14 ± 0.015	− 22 ± 1.12	46.7 ± 0.10

### In vitro release study

**Fig. 3:**
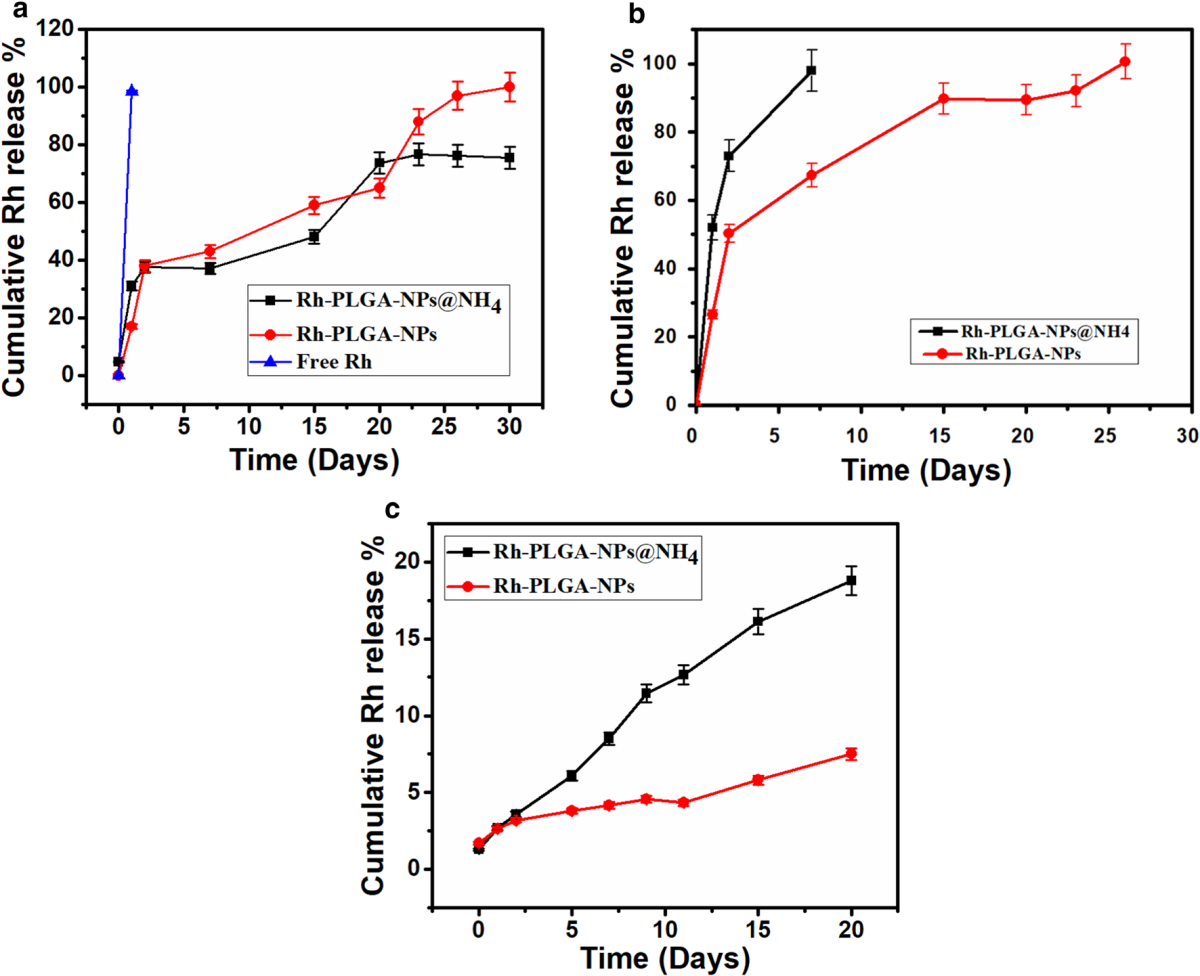
In-vitro release of Rh from Rh-PLGA-NPs@NH4 and Rh-PLGA-NPs@NH4 in dissolution media phosphate buffer, at different **a** pH 7.4 **b** pH 5.4) and **c** SFE. Data are means ± S.D., n = 03

The Rh release from Rh loaded in Rh-PLGA-NPs@NH_4_ and Rh-PLGA-NPs at different pH (7.4 and 5), simulating mixture of the SFE in OA were shown in Fig. 3 (Farr et al. 1985).

The final result clearly revealed that pH has a significant role in the in vitro release. The cumulative release profile of Rh at 7.4 pH were comparable for all the prepared NPs, whereas free Rh was completely released within 24 h (Fig. 3a). However, at pH 5 a completely different release pattern was observed in which we found that 72% of Rh was released from Rh-PLGA-NPs@NH_4_ within 48 h whereas complete release was obtained on seventh day of the study. The Rh release from Rh-PLGA-NPs have a sustained pattern contrast to that of Rh-PLGA-NPs@NH_4_ where only 67% of the Rh was release from Rh-PLGA-NP up to seventh day as show in Fig. 3b. At last in SFE, the released profile of Rh from Rh-PLGA-NPs@NH_4_ showed a faster release rate as compared to other NPs at the 5th day (Fig. 3c) revealing the similar effect as in acidic release media.

### Cell viability assay

**Fig. 4:**
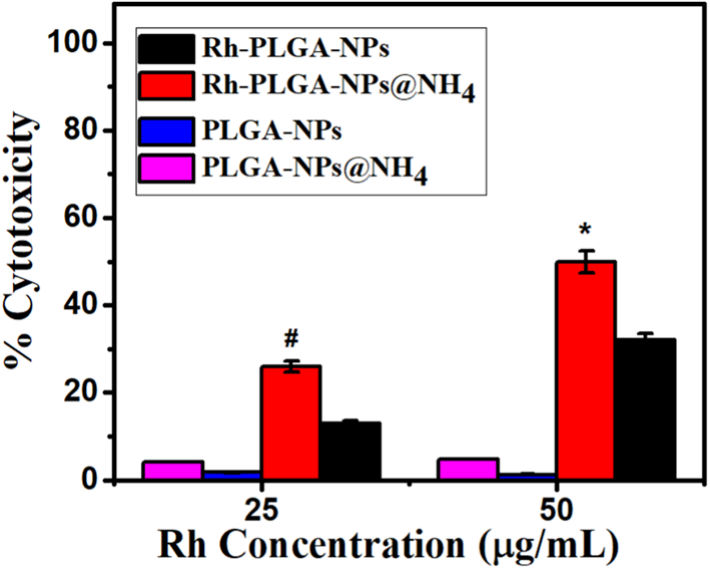
% Cell viability of THP-1 cell exposed to concentrations 25 µg/ mL and 50 µg/mL of control, Rh-PLGA-NPs and Rh-PLGA-NPs@NH_4_ (n = 06). (*p < 0.01; Rh-PLGA-NPs@NH_4_ vs. Rh-PLGA-NPs, # p < 0.05; Rh-PLGA-NPs@NH_4_ vs. Rh-PLGA-NPs)

The biocompatibility of Rh-PLGA-NPs@NH_4_ and other developed NPs was calculated by using MTT assay. The THP-1 cell lines were treated at different equivalent concentration as shown in (Fig. 4). The cytotoxicity results revealed increase in cytotoxicity with increase in the concentration of Rh. Rh-PLGA-NPs@NH_4_ exhibited a slightly higher cytotoxicity (p < 0.05) compared with Rh-PLGA-NPs at 25 µg/ mL after 24 h of incubation. However, at higher drug concentrations 50 µg/ mL, Rh-PLGA-NPs@NH_4_ indicates significant (p < 0.01) decrease in cell viability than Rh-PLGA-NPs. The Rh-PLGA-NPs@NH_4_ were intended for treatments in chronic patients, therefore the high concentration cytotoxicity could be discarded (Campos et al. 2013). Further, there was negligible cytotoxicity was observed for PLGA-NPs, PLGA-NPs@NH_4_.

### Rh-PLGA-NPs@NH4 uptake studies

**Fig. 5:**
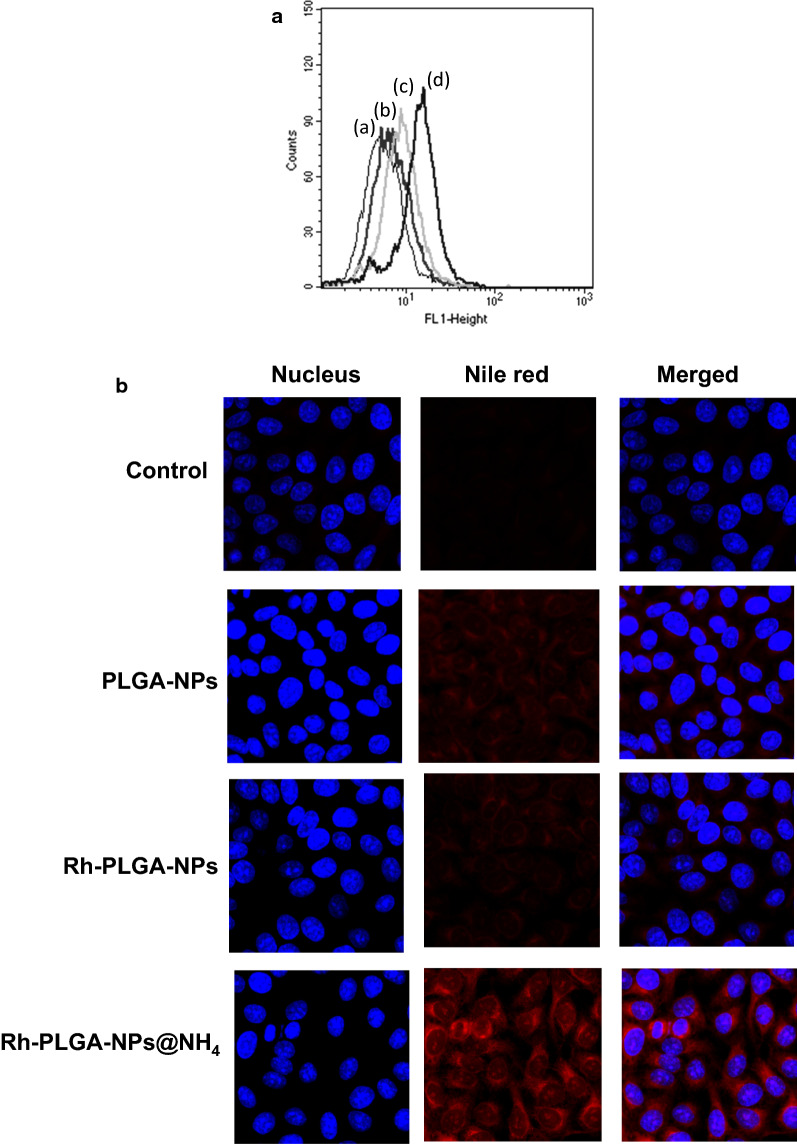
Intracellular uptake of Nile red encapsulated PLGA-NPs, Rh-PLGA-NPs and Rh-PLGA-NPs@NH4 **a** flow cytometer analysis **b** confocal microscope

Next, we performed the Rh-PLGA-NPs@NH_4_ uptake studies and compare the results with the other developed particles. The study was conducted in THP-1 cells when Rh-PLGA-NPs@NH_4_ along with other particles were incubated for 4 h with the cells. The results were verified by flow cytometer analysis and confocal microscope. The flow cytometer analysis results revealed that the Rh-PLGA-NPs@NH_4_ were well engulfed in the cells. However, other particles also engulfed by the cells, which might be attributed to the size of particles. Further, the images obtained from confocal microscopy confirmed the uptake pattern of Rh-PLGA-NPs@NH_4_. The higher release of Rh from pH-sensitive Rh-PLGA-NPs@NH_4_ resulted in higher cell uptake in comparison with the non-pH-sensitive NPs. In the cellular engulfment, particle size of NPs plays an important role and the size of Rh-PLGA-NPs@NH_4_ was optimum for cellular uptake. Also, the hydrophobic NPs could be embedded in inner hydrophobic core of the bilayers by directly penetrating into the cellular membrane. The results have been illustrated in Fig. 5a, b for flow cytometer analysis and confocal microscope respectively.

### Anti-inflammatory cytokine assay and ROS generation

**Fig. 6:**
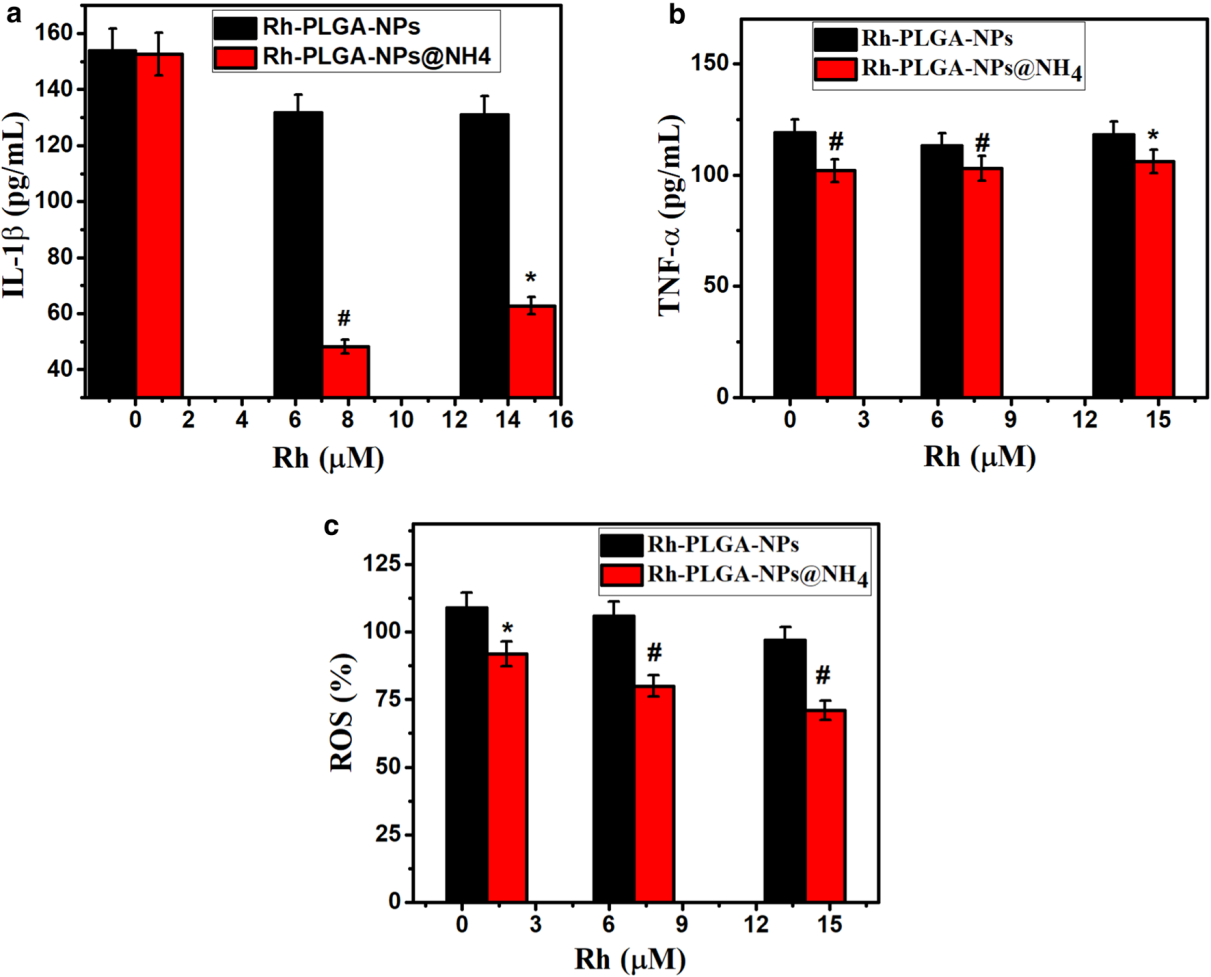
PLGA-NPs and Rh-PLGA-NPs@NH_4_ effect on **a** IL-1β (*p < 0.01; Rh-PLGA-NPs@NH_4_ vs. Rh-PLGA-NPs, #p < 0.001; Rh-PLGA-NPs@NH_4_ vs. Rh-PLGA-NPs). **b** TNFα (*p < 0.05; Rh-PLGA-NPs@NH_4_ vs. Rh-PLGA-NPs, #p < 0.05; Rh-PLGA-NPs@NH_4_ vs. Rh-PLGA-NPs) and **c** ROS levels (*p < 0.05; Rh-PLGA-NPs@NH_4_ vs. Rh-PLGA-NPs, #p < 0.01; Rh-PLGA-NPs@NH_4_ vs. Rh-PLGA-NPs). Data represent the mean ± SD (N = 03)

The final result of Rh-PLGA-NPs@NH_4_ on the generation of IL-1β, TNF-α and ROS were illustrated in Fig. 6a–c). As revealed in the curve, there was significant inhibition of IL-1β (p < 0.01) and ROS (p < 0.01) after the treatment with Rh-PLGA-NPs@NH_4_ as compared with Rh-PLGA-NPs as control group. The slightly less reduction in the results of TNF-α (p < 0.05) was observed for Rh-PLGA-NPs@NH_4_ as compared with Rh-PLGA-NPs. This might be attributed to the release kinetics of Rh. A significant reduction in the level of cytokine even at the lower concentration of Rh in Rh-PLGA-NPs@NH_4_ was observed in comparison with Rh-PLGA-NPs. The results were similar to the previous findings by Gómez-Gaete et al. (2017).

The generation of intracellular ROS is one of the factors responsible for the inflammation in OA. Accumulation of ROS is directly associated with oxidative stress and production of ROS and thus the inflammatory response. Rh-PLGA-NPs@NH_4_ could reduce ROS stress in cells compared to the control group (Gao et al. 2014). Moreover, exposure of Rh-PLGA-NPs@NH_4_ induced the advanced levels of intracellular ROS compared to that control (Fig. 6c). The in vitro results obtained from the above studies clearly reveals the potential of developed Rh-PLGA-NPs@NH_4_ in reducing the inflammation. The treatment of OA still offers a huge challenge due to local and systemic adverse effect by giving long term treatment. The pH sensitive Rh loaded Rh-PLGA-NPs@NH_4_HCO_3_ was successfully prepared for effective nano-therapy in OA. The developed Rh-PLGA-NPs@NH_4_HCO_3_ release Rh in controlled manner and significantly reduces the inflammatory cytokines in the in vitro studies. The results could be concluded that the developed particles could be further used in the in vivo model of OA to verify its efficacy.

## Discussion

OA has been characterized as a disease in which pro-inflammatory cytokine plays an important role (Sokolove et al. 2013). Damage of cartilages, bones and inflamed synovial tissues could be commonly observed in OA. Rh has been known to act on pro-inflammatory cytokine particularly on IL-1β and TNF-α which are present in synovial fluid of knee join in high concentration of OA patients (Mabey et al. 2015). Therefore, in the present work we have successfully developed the pH-sensitive Rh-PLGA-NPs@NH_4_. The pH-sensitive NPs could provide the effective release of Rh at the lower pH of synovial fluid (Li et al. 2017). The Rh-PLGA-NPs@NH_4_ were developed using double emulsion method, this method provides the encapsulation of the drug on the shell of NPs optimum for the burst release. The size of the NPs could also be controlled in double emulsion method (McCall et al. 2013). The size of the Rh-PLGA-NPs@NH_4_ was slightly greater than Rh-PLGA-NPs as observed by DLS and SEM studies. The NPs developed have smooth surface as observed in the SEM images which might be attributed to the PVA used as an emulsifier. However, the size of both Rh-PLGA-NPs@NH_4_ and Rh-PLGA-NPs were optimum for cell engulfment (Behzadi et al. 2017). The size of the NPs have high significance in the treatment as the greater size could trigger the immune response and might also induce swelling at the particular site. Also, the surface charge of the particles was − 22 ± 1.12 mV which has been established as appropriate value of a stable formulation as it could avoid the aggregate formation of the particles in a dispersion state. It was observed that, the encapsulation efficiency of Rh was reduced in Rh-PLGA-NPs@NH_4_ when compared to Rh-PLGA-NPs which might be due to the loading of NH_4_HCO_3_ along with Rh. The release study results clearly revealed the significant role of pH, where no much difference at 7.4 pH was observed for developed Rh-PLGA-NPs@NH_4_ and Rh-PLGA-NP. However, at a slightly acidic medium pH 5 a burst release was observed from Rh-PLGA-NPs@NH_4_ in comparison to Rh-PLGA-NPs. The results might be attributed to the porous nature of PLGA which allows the H_3_O^+^ in Rh-PLGA-NPs@NH_4_ which reacts with NH_4_HCO_3_ and produces NH_4_^+^, CO_2_ and H_2_O (Zerrillo et al. 2019). The reaction increase the rupture of Rh-PLGA-NPs@NH_4_ due to high pressure resulting in burst release of Rh. Also, initial release of the drug suggests that the fraction of the drug might be encapsulated in the shell of the NPs. Further, NPs without Rh did not revealed any significant cytotoxicity to the cells as of biocompatible PLGA was used to deliver Rh (Elmowafy et al. 2019). However, the results obtained from the cytotoxicity studies conducted on THP-1 cell lines clearly revealed the Rh concentration based reduction in the cell viability. As we intend to administer this dose to the patients with chronic disease, the toxicity associated with high concentration could be discarded. The results obtained for the anti-inflammatory effect of Rh-PLGA-NPs@NH_4_ on LPS stimulated THP-1 cells revealed the significant reduction of the pro-inflammatory cytokines (Gómez-Gaete et al. 2017). The results might be attributed to the inhibition of NF-κβ by Rh, responsible for the production of IL-1β due to LPS stimulation (Ge et al. 2017). The ROS production is often associated with the joint inflammation and also responsible for oxidative stress. The reduction in ROS could be attributed to the inhibition of the release of pro-inflammatory cytokines.

The results of in-vitro studies revealed that the pH sensitive Rh-PLGA-NPs@NH_4_ potentially inhibit the pro-inflamatory cytokines and ROS in LPS stimulated THP-1 cells. Therefore, it could be stated that the in-vivo animal studies might find the advantage from this kind of delivery systems in OA. The findings of the above study demonstrates the great potential of Rh-PLGA-NPs@NH_4_ for the treatment of OA. However, it could be suggested that the pH-sensitive NPs could be mixed with non-pH-sensitive NPs to improve the initial release of Rh that could enhance therapeutic potential at the early stage of treatment.

In summary, we have successfully developed pH-sensitive Rh loaded Rh-PLGA-NPs@NH_4_ using a double emulsion method for the effective treatment of OA. The particles were well characterized and their response in different pH-environment was evaluated as well as their in-vitro efficacy on inflammatory cytokines were evaluated. The results revealed that Rh-PLGA-NPs@NH_4_ was sensitive for low pH and offers burst release and significantly reduces the inflammatory cytokines in the in vitro studies. In conclusion it might be stated that Rh-PLGA-NPs@NH_4_ could provide therapeutic solution to those patients who suffer from chronic joint ailments by reducing the progression of OA. It also suggest that these particles may be an effective method for developing vaccination.
